# Supplementary material for: Ice Regelation: Hydrogen-bond extraordinary recoverability and water quasisolid-phase-boundary dispersivity
Source: Sci Rep. 2015 Sep 9;5:13655. doi: 10.1038/srep13655 (PMC4563362; doi:10.1038/srep13655)
Supplement: Supplementary Information [file srep13655-s1.docx]

**Supporting Information:**

**Ice Regelation: Hydrogen-bond extraordinary recoverability and water quasi-solid-phase-boundary dispersivity**

Xi Zhang,^1,2#^ Yongli Huang,^3#^ Peng Sun^1#^, Xinjuan Liu,^1,*^ Zengsheng Ma,^3^ Yichun Zhou,^3^ Ji Zhou,^4^ and Weitao Zheng^5^, Chang Q Sun,^6,^^[[1]](#footnote-1)^

1. *Institute of Coordination Bond metrology and Engineering, College of Materials Science and Engineering, China Jiliang University, Hangzhou 310018, China*
2. *Institute of Nanosurface Science and Engineering, Shenzhen University, Shenzhen 518060, China*
3. *Key Laboratory of Low-dimensional Materials and Application Technology (MOE) and School of Materials Science and Engineering, Xiangtan University, Xiangtan, 411105, China*
4. *State Key Laboratory of New Ceramics and Fine Processing, Department of Materials Science and Engineering, Tsinghua University, Beijing 100084, China*
5. *School of Materials Science, Jilin University, Changchun 130012, China*
6. *NOVITAS, School of Electrical and Electronic Engineering, Nanyang Technological University, Singapore 639798*

The MD calculations were performed using Forcite’s package with *ab initio* optimized forcefield Compass27.[[1](#_ENREF_1)] The Compass27 has been widely used in dealing with the electronic structures and the hydrogen bond network of water and amorphous ices[[2](#_ENREF_2), [3](#_ENREF_3)] as well as water chains in hydrophobic crystal channels.[[4](#_ENREF_4)] Ice-VIII consisting of two interpenetrating cubic ice lattices, of 8 molecules in each unit cell is examined. The MD calculations were performed to examine the evolution of the O-H and O : H distances in a 2×2×1 supercell of ice-VIII unit, containing 32 molecules, under the pressure changing from 1 to 20 GPa. The structure was dynamically relaxed under the Isoenthalpic–isobaric ensemble for 30 ps, showing sufficiently stable convergence. The average O-H and O : H lengths were taken of the structures of the last 10 ps (20,000 steps).

References:

1. H. Sun, *COMPASS: An ab Initio Forcefield Optimized for Condensed-Phase Applications - Overview with Details on Alkane and Benzene Compounds.* **J. Phys. Chem. B,** 1998. **102**: 7338.

2. C. He, J.S. Lian, and Q. Jiang, *Electronic structures and hydrogen bond network of ambient water and amorphous ices.* **Chem. Phys. Lett.,** 2007. **437**(1-3): 45-49.

3. J.B. Cao and J.Q. Wu, *Strain effects in low-dimensional transition metal oxides.* **Materials Science & Engineering R-Reports,** 2011. **71**(2-4): 35-52.

4. R. Natarajan, J.P.H. Charmant, A.G. Orpen, and A.P. Davis, *Water Chains in Hydrophobic Crystal Channels: Nanoporous Materials as Supramolecular Analogues of Carbon Nanotubes.* **Angewandte Chemie-International Edition,** 2010. **49**(30): 5125-5129.

1. #Authors made equal contribution

   ‘ Corresponding Author: [Liuxijuan@cjlu.edu.cn](mailto:Liuxijuan@cjlu.edu.cn); [ecqsun@ntu.edu.sg](mailto:ecqsun@ntu.edu.sg) [↑](#footnote-ref-1)
